# Exosomes released from pancreatic cancer cells enhance angiogenic activities via dynamin-dependent endocytosis in endothelial cells *in vitro*

**DOI:** 10.1038/s41598-018-30446-1

**Published:** 2018-08-10

**Authors:** Mitsuru Chiba, Shiori Kubota, Konomi Sato, Satoru Monzen

**Affiliations:** 1 Department of Bioscience and Laboratory Medicine, Graduate School of Health Sciences, Hirosaki University, 66–1 Hon-cho, Hirosaki, Aomori, 036–8564 Japan; 2 Department of Medical Technology, School of Health Sciences, Hirosaki University, 66–1 Hon-cho, Hirosaki, Aomori, 036–8564 Japan; 3 Department of Radiation Science, Graduate School of Health Sciences, Hirosaki University, 66–1 Hon-cho, Hirosaki, Aomori, 036–8564 Japan

## Abstract

Pancreatic cancer has the lowest 5 year survival rate among all cancers. Several extracellular factors are involved in the development and metastasis of pancreatic cancer to distant organs. Exosomes are lipid-bilayer, membrane-enclosed nanoparticles that are recognised as important mediators of cell-to-cell communications. However, the role of exosomes released from pancreatic cancer cells in tumour micro-environment remains unknown. Here, we show that exosomes released from pancreatic cancer PK-45H cells activate various gene expressions in human umbilical vein endothelial cells (HUVECs) by *in vitro* analyses. In addition, these exosomes released from PK-45H cells promote phosphorylation of Akt and ERK1/2 signalling pathway molecules and tube formation via dynamin-dependent endocytosis in HUVECs. Our findings suggested that exosomes released from pancreatic cancer cells may act as a novel angiogenesis promoter.

## Introduction

Pancreatic cancer is the deadliest major cancer with a 5 year survival rate of 8% in the United States^1^. This is probably because subjective symptoms in pancreatic cancer are poorly recognised, although progression is remarkably rapid with early metastasis and peritoneal dissemination. Invasive pancreatic cancers from normal pancreatic ductal cells arise mostly from pancreatic intraepithelial neoplasms. Genetic mutation of the *K-Ras* oncogene is present in 95% of pancreatic ductal adenocarcinomas. *K-Ras*^*G12D*^ mutation activates and stimulates signalling pathways to induce cell proliferation, invasion and metastasis^2^. Inactivation by genetic mutation of tumour-suppressor genes (*CDKN2A*, *TP53*, *SMAD4* and so forth) also is involved in progression of pancreatic cancer^2^. Pancreatic cancer cells that accumulate these genetic mutations metastasise to vessels, lymph nodes, liver and other distant organs. As a result, most patients are initially diagnosed with disease progression and have minimal or no possibility for radical cure.

The tumour tissues are composed of cancer, normal, endothelial, fibroblast, mesenchymal stem and immune cells^3^. Cell-to-cell communications occurring via several extracellular factors, such as vascular endothelial growth factor (VEGF), fibroblast growth factor, matrix metalloproteases, transforming growth factor-β, platelet-derived growth factor, chemokines and cytokines, released from these cells contribute to development and metastasis of cancer cells in the tumour micro-environment^4^. For example, VEGF, which is a growth factor, activates the classical mitogen-activated protein (MAP) kinase and phosphatidylinositol-3 kinase (PI3K)/Akt pathways in endothelial cells with VEGF receptor 2, enhances angiogenesis and induces metastasis of cancer cells^4,5^. Multiple mechanisms via various extracellular factors are involved in angiogenesis and metastasis. Therefore, further information is needed to understand mechanisms of angiogenesis and metastasis in the tumour micro-environment.

Exosomes were first observed approximately three decades ago in differentiating reticulocytes^6^. Exosomes are extracellular vesicles (40–200 nm in diameter) that are constitutively released from most cell types, including cancer cells^7^. Recently, exosomes released from pancreatic cancer cells were reported to be involved in metastasis of cancer cells to distant organs. Yu *et al*. indicated that exosomes released from highly metastatic pancreatic cancer cells promoted tumour growth and liver metastasis^8^. The cell-to-cell communications between cancer and stromal cells via exosomes have a pivotal role in cancer progression and metastasis. Masamune *et al*. reported that exosomes released from pancreatic cancer cells activate PI3K/Akt and MAP kinase signalling in pancreatic stellate cells^9^. However, the effects on endothelial cells in exosomes released from pancreatic cancer cells remain unknown. Angiogenesis has been reported to be enhanced in chronic pancreatitis and pancreatic cancer^10^. but studies on angiogenesis via exosomes are few. We focused on exosomes released from pancreatic cancer cells, which is a known extracellular factor, and investigated their effects on signalling molecules, gene expressions and phenotypes of endothelial cells that form part of the tumour environment by *in vitro* analysis.

## Results

### Detection of exosomes released from PK-45H cells

**Figure 1:**
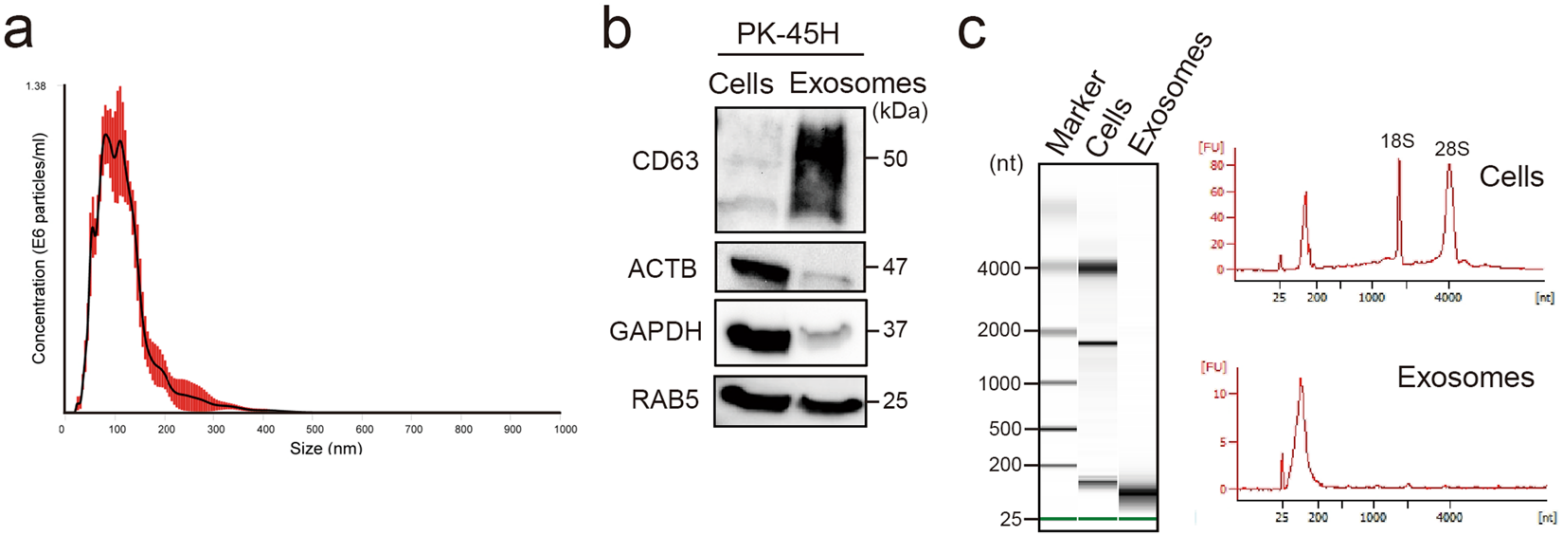
Detection of exosomes and exosomal proteins and RNAs released from PK-45H cells. (**a**) Detection of particles in culture supernatants of PK-45H cells. The *horizontal axis* represents the particle size (nm), whereas the *vertical axis* represents the particle concentration (×10^6^ particles/mL). *Red bar* represents the standard errors of means. The mean particle size is 115 ± 9.1 nm. (**b**) Detection of CD63, ACTB, GAPDH and RAB5 proteins in the exosomes released from PK-45H cells and the cellular proteins by Western blotting. CD63, a tetraspanin family member, known as the exosomal marker, was detected in the exosomal proteins. Full-length blots are present in Supplementary Fig. S2. (**c**) Detection of exosomal RNAs released from PK-45H cells and the cellular RNAs using the Agilent 2100 Bioanalyzer. The peak detected 25 nucleotides (nt), representing an internal standard. The peak of small RNAs (25–200 nt) was detected in the exosomal RNAs. FU, fluorescence units.

To confirm the size of exosomes released from pancreatic cancer PK-45H cells (derived from liver metastasis), exosomal particles in the culture supernatants were collected and examined using the NanoSight LM10 (NanoSight; Malvern, UK). Mean particle diameter was 115 ± 9.1 nm (Fig. 1a), so the particles were characterised as exosomes.

Exosomes are known to contain various RNAs and proteins, especially the tetraspanin family^7^. To detect cellular and exosomal proteins, Coomassie Brilliant Blue staining was performed, which showed that exosomes released from PK-45H cells contained several protein bands, albeit in lower counts than those released from cellular proteins (Supplementary Fig. S1). An exosomal marker (CD63), housekeeping markers (ACTB and GAPDH) and an endosome marker (RAB5) were examined in cells and exosomes released from PK-45H cells by Western blotting. CD63 was highly detected in exosomal proteins, and ACTB and GAPDH were detected mainly in cellular proteins. RAB5 was detected in both proteins (Fig. 1b, Supplementary Fig. S2). To confirm whether exosomes released from PK-45H cells and whether the cellular proteins contained RNAs, RNAs were extracted with the Isogen II reagent. The size of cellular and exosomal RNAs was examined with the Agilent 2100 Bioanalyzer (Agilent Technologies, Foster City, CA, USA). Peaks of 18 S ribosomal RNAs (rRNA) and 28 S rRNAs were detected in cellular RNAs. On the other hand, peaks of small RNAs (25–200 nucleotides) were detected in exosomal RNAs, although peaks of rRNA were not detected (Fig. 1c). These observations indicated that particles collected by ultracentrifugation contained numerous exosomes released from PK-45H cells.

### Exosomes released from PK-45H cells are taken up by human umbilical vein endothelial cells (HUVECs) via dynamin-dependent endocytosis

To confirm the characteristics of endothelial cells in HUVECs, reverse-transcription quantitative polymerase chain reaction was performed using total RNAs extracted from HUVECs. The mRNAs of endothelial markers (*CD31*, *CD105*, *CD144*, *VEGFR1*, *VEGFR2* and *vWF*) were expressed in used HUVECs (Supplementary Fig. S3).

**Figure 2:**
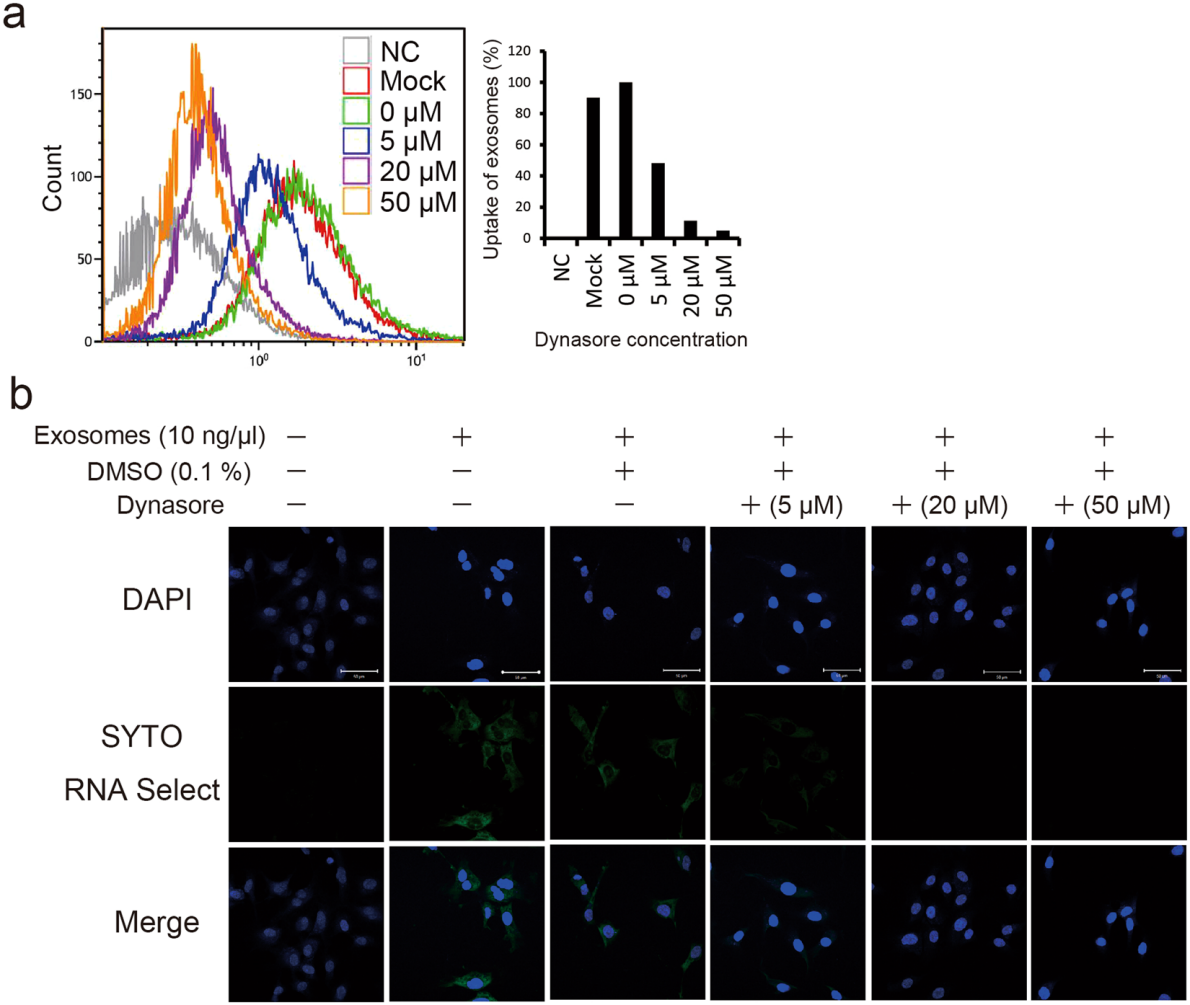
Uptake of exosomes released from PK-45H cells into HUVECs. (**a**) Effects on the uptake of exosomes by dynasore treatment using the FACS Aria. Exosomes were stained by the SYTO RNA Select Reagent and added to the culture media of dynasore-treated or untreated HUVECs. Treatment with >5 µM dynasore inhibited exosome uptake. (**b**) Effects on exosome uptake at 3 h after dynasore treatment observed under a confocal laser scanning microscope (LSM710; Carl Zeiss). Nucleus was stained by DAPI. *Scale bar*: 50 µm.

Dynamin is a GTPase protein essential for membrane fusion during clathrin-mediated endocytosis in eukaryotic cells^11^. Dynasore is a small, non-selective inhibitor of the GTPase activity of dynamin^12^. Exosomes have been reported to be taken up by dynamin-mediated endocytosis^13^. To investigate uptake of exosomes released from PK-45H cells into HUVECs, exosomes stained with SYTO RNA Select Reagent (Thermo Fisher Scientific, Carlsbad, CA, USA) were added to the culture media of dynasore-treated or untreated HUVECs and analysed using the BD FACS Aria (Becton Dickinson, Franklin Lakes, NJ, USA). When 10 ng/µL exosomes stained with the SYTO RNA Select Reagent were treated with >5 µM dynasore to HUVECs, uptake of exosomes decreased conspicuously (Fig. 2a). In addition, 10 ng/µL stained exosomes was added to the culture media of dynasore-treated or untreated HUVECs to examine their uptake by confocal laser scanning microscopy. Green fluorescence was detected in PK-45H cells at 3 h after addition of SYTO RNA Select-stained exosomes, and the decrease in green fluorescence depended on the dynasore concentration treated in HUVECs (Fig. 2b). These results suggested that exosomes released from PK-45H cells may be taken up in HUVECs via dynamin-dependent endocytosis.

### Exosomes released from PK-45H cells induce changes in the gene expressions to HUVECs

To examine gene expression in HUVECs treated with exosomes released from PK-45H cells, microarray analyses were performed using the SurePrint G3 Human GE Microarray (Agilent Technologies). Total RNAs from HUVECs treated or not with 10 ng/µL of exosomes released from PK-45H cells for 48 h were extracted using Isogen II reagent (NipponGene, Tokyo, Japan). The >1.50–fold up- and down-regulated probes in treated HUVECs were 7203 and 3594, respectively (Supplementary Table S1). This result indicated that gene expression changed in the treated HUVECs. To predict activated or inhibited molecules and signalling pathways in treated HUVECs, Ingenuity Pathway Analysis (IPA; Qiagen, Redwood City, CA, USA) was performed using the up- and down-regulated probes. Activated or inhibited signalling in treated HUVECs was predicted by the ‘upstream analysis’ of the IPA system. The upstream regulators of 67 ‘Activated (*z*-score > 2.000)’ and 26 ‘Inhibited (*z*-score < −2.000)’ were detected (Supplementary Table S2). The typical regulators among these are presented in Table 1. Activations of HRAS, VAGFA, AKT and ERK1/2 and inhibitions of U0126 and Sulforaphane were predicted by the ‘upstream analysis’ (Table 1), suggesting the activations of PI3K/Akt and MAP kinase signalling pathways.

**Table 1:** Prediction of activated/inhibited signalling in HUVECs treated with exosomes released from PK-45H cells.

Upstream regulator	Molecule type	Predicted activation state	Activation *z*-score
PDGF BB	Complex	Activated	2.881
HRAS	Enzyme	Activated	2.739
VAGFA	Growth factor	Activated	2.529
HSP90	Group	Activated	2.488
EDN1	Cytokine	Activated	2.462
TRAF6	Enzyme	Activated	2.407
AKT	Group	Activated	2.273
ERK1/2	Group	Activated	2.118
U0126	Chemical–kinase inhibitor	Inhibited	−2.485
Sulforaphane	Chemical drug	Inhibited	−2.747

**Figure 3:**
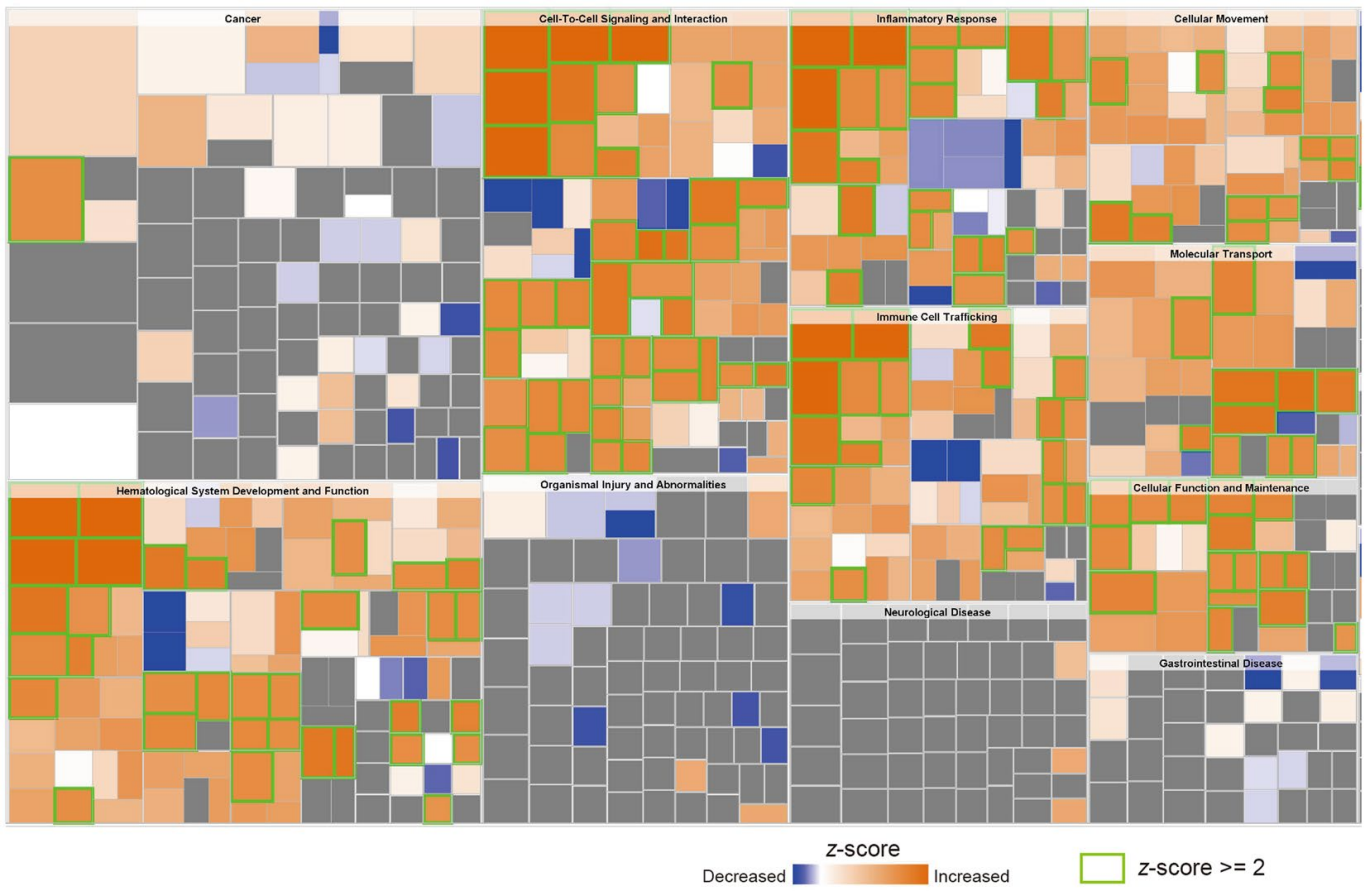
Prediction of functions in HUVECs treated with exosomes released from PK-45H cells. The effects on the functions in treated HUVECs were examined by analysis of the ‘disease and functions’ of the IPA system. Size and colour are shown by the −log (*P*-value) or *z*-score, respectively. *Orange* and *blue* colours in the heat map indicate higher and lower *z*-scores, respectively. *Green scale* represents ‘Increased (*z*-score > 2.000)’ functions. The category data for 75 ‘increased (*z*-score > 2.000)’ and 1 ‘decreased (*z*-score < −2.000)’ are shown in Supplementary Table S3. The category of ‘cell-to-cell signalling and interaction’ included a number of ‘increased (*z*-score > 2.000)’.

Furthermore, the effects on the functions in treated HUVECs were examined by analysis of ‘disease and functions’ using IPA and microarray data. The ‘activation *z*-score’ and ‘predicted activation state’ in all ‘categories’ of function were analysed by IPA. The categories of 75 ‘increased (*z*-score > 2.000)’ and 1 ‘decreased (*z*-score < −2.000)’ were predicted in the ‘predicted activation state’ (Supplementary Table S3). On the basis of the above information, a heat map was created predicting that categories of ‘haematology system development and function’, ‘cell-to-cell signalling and interaction’, ‘inflammatory response’, ‘immune cell tracking’, ‘cellular movement’, ‘molecular transport’ and ‘cellular function and maintenance’ activated in this order (Fig. 3). The category of ‘cell-to-cell signalling and interaction’ especially included a number of ‘increased (*z*-score > 2.000)’. These findings predicted that exosomes released from PK-45H cells may activate several signalling molecules involved in the cell survival and mortality of HUVECs via cell-to-cell communications.

### Effects on signal molecules in HUVECs treated with exosomes released from PK-45H cells

**Figure 4:**
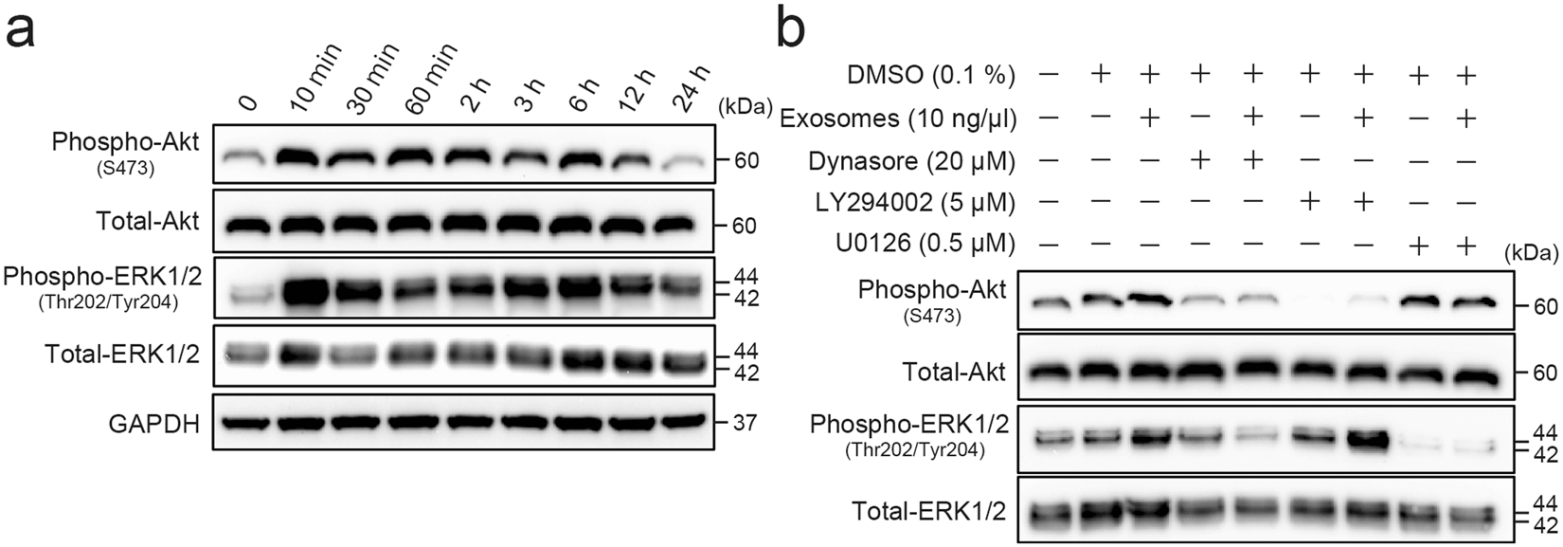
Activations of PI3K/Akt and MAP kinase signalling in HUVECs treated with exosomes released from PK-45H cells. (**a**) Phosphorylation status of Akt and ERK1/2 in treated HUVECs. Phosphorylated proteins were detected by Western blotting. Treatment of exosomes enhanced phosphorylation of Akt and ERK1/2 in HUVECs. Full-length blots are present in Supplementary Fig. S4. (**b**) Effects on activation of Akt and ERK1/2 in treated HUVECs in the presence or absence of dynasore, PI3 kinase inhibitor (LY294002) and MEK inhibitor (U0126). Phosphorylation of Akt and ERK1/2 was induced by dynamin-dependent exosomal endocytosis. Full-length blots are present in Supplementary Fig. S5.

Activations of PI3K/Akt and MAP kinase signalling were predicted in treated HUVECs (Table 1). Therefore, the phosphorylation status of Akt and ERK1/2 in HUVECs treated with 10 ng/µL exosomes was examined by Western blotting. Exosomes induced increased expressions of phospho-Akt (S473) and phosphor-ERK1/2 (Thr202/Tyr204) in HUVECs within 10 min (Fig. 4a, Supplementary Fig. S4). Phosphorylation of Akt and ERK1/2 continued at least for 12 h after treatment with 10 ng/µL exosomes (Fig. 4a, Supplementary Fig. S4). This indicated that exosomes released from PK-45H cells early lead to activation of PI3K/Akt and MAP kinase signalling pathways in HUVECs.

To examine whether the uptake of exosomes released from PK-45H cells induced activation of Akt and ERK1/2, the expressions of phospho-Akt (S473) and phosphor-ERK1/2 (Thr202/Tyr204) in HUVECs treated with 10 ng/µL exosomes for 10 min with or without dynasore, PI3 kinase inhibitor (LY294002) and MEK inhibitor (U0126) were examined. Phosphorylation of Akt and ERK1/2 in treated HUVECs increased in the absence of 20 μM dynasore and was inhibited in the presence of 20 μM dynasore (Fig. 4b, Supplementary Fig. S5). In addition, Akt or ERK1/2 phosphorylation in treated HUVECs was inhibited in the presence of 5 μM LY294002 or 0.5 μM U0126, respectively (Fig. 4b, Supplementary Fig. S5). These results suggested that phosphorylation of Akt and ERK1/2 may be induced by uptake of exosomes via dynamin-dependent endocytosis.

### Effects on tube formation in HUVECs treated with exosomes released from PK-45H cells

**Figure 5:**
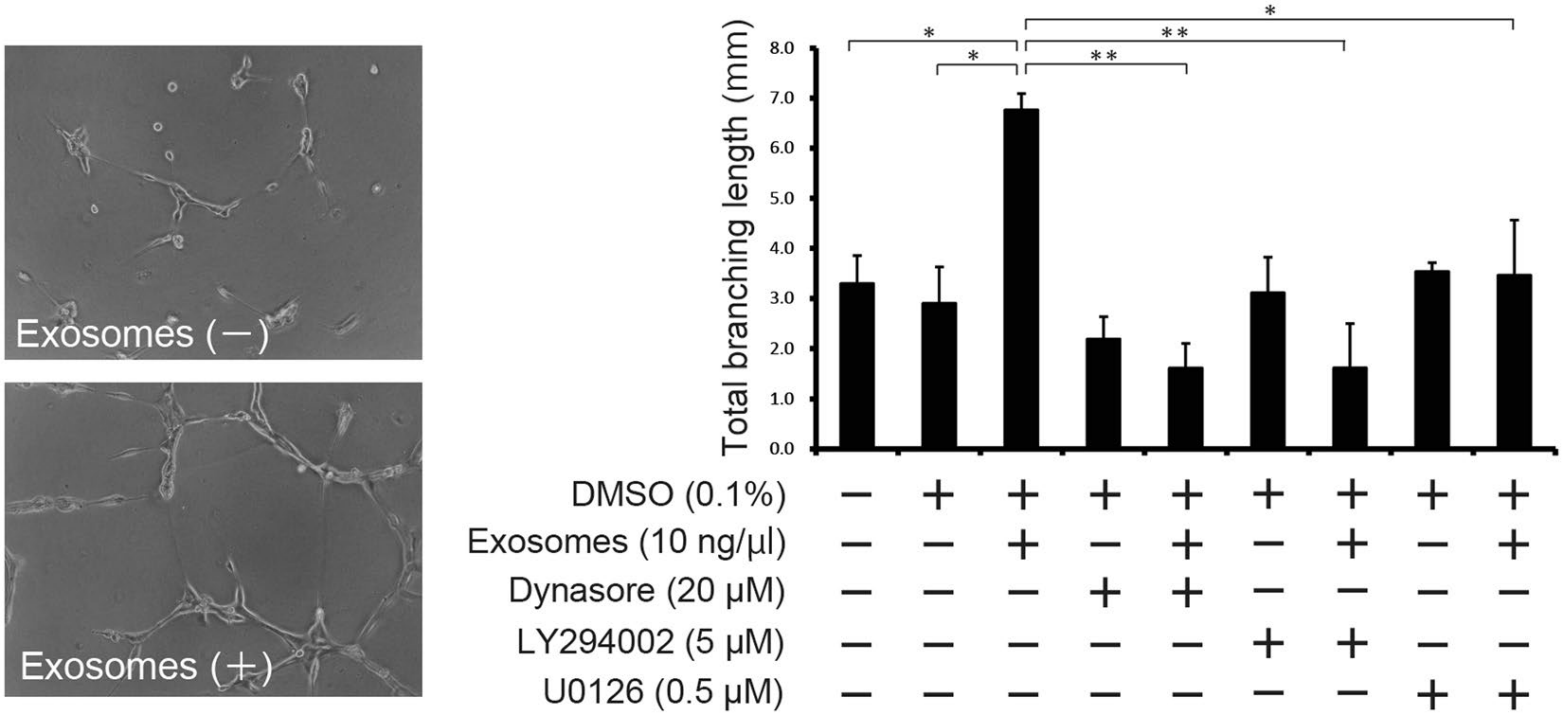
Effects on tube formation in HUVECs treated with exosomes released from PK-45H cells. Tube formation assay was performed using the Cultrex *In Vitro* Angiogenesis Assay Tube Formation Kit (Cosmo Bio). A dynamin-dependent exosomal endocytosis promoted tube formation in HUVECs. Data are expressed as means ± standard deviations (each *n* = 3). Statistical significance (*P* ≤ 0.05) was examined with the Tukey–Kramer test for multiple comparisons.

The categories of ‘cell-to-cell signalling and interaction’ and ‘cellular movement’ strongly correlated with analysis of ‘disease and functions’ using IPA (Fig. 3). Therefore, we examined the effects on the capacity of tube formation in HUVECs after exosome treatment. We measured the total branching lengths in HUVECs treated with 10 ng/µL exosomes for 9 h with or without 20 μM dynasore, 5 μM LY294002 or 0.5 μM U0126. Concentrations of 20 μM dynasore, 5 μM LY294002 or 0.5 μM U0126 do not induce serious cell toxicity (Supplementary Fig. S6). Total branching length increased significantly with exosome treatment (*P* ≤ 0.05) and were suppressed significantly in HUVECs treated with exosomes in the presence of dynasore, LY294002 and U0126 (*P* ≤ 0.01, *P* ≤ 0.01 and *P* ≤ 0.05, respectively; Fig. 5). These results indicated that uptake of exosomes via dynamin-dependent endocytosis promotes tube formation in HUVECs *in vitro*.

## Discussion

We demonstrated that exosomes released from pancreatic cancer cells could promote tube formation in HUVECs *in vitro*. In addition, uptake of exosomes via dynamin-dependent endocytosis might enhance phosphorylation of the Akt and ERK1/2 signalling pathways and changed the expressions of several genes *in vitro*. This observation suggested that exosomes released from pancreatic cancer cells may enhance angiogenesis in primary tumour and micrometastasis in distant organs.

Blood vessels are essential for tumour growth as a means of obtaining nutrients and oxygen, as well as for evacuating metabolic wastes. Angiogenesis is an important vital phenomenon in the tumour micro-environment to maintain tumour expansion in a short time, especially in advanced-stage cancer. VEGF is a widely accepted proangiogenic factor that is up-regulated when stimulated by hypoxia or oncogene signalling^14^. Similarly, other proangiogenic genes, such as fibroblast growth factor and matrix metalloproteinase 9, also are up-regulated in tumourigenesis^15^. Angiogenesis is activated by various cell signalling pathways that induce dramatic changes in gene expressions during tumour progression. The networks of these molecules and mechanisms involved in cell-to-cell communication are extremely complex. Recently, exosomes released from cancer cells were reported to be important tools in cell-to-cell communications and to have important roles in the angiogenesis of tumour micro-environment, haematogenous metastasis and engraftment of distant organs^13,16–22^. For example, Grande *et al*. reported that microvesicles released from human renal cancer stem cells conferred an activated angiogenetic phenotype to normal human endothelial cells^18^. On the other hand, Mineo *et al*. reported that exosomes released from K562 chronic myeloid leukaemia cells induced phosphorylation of Akt and ERK1/2 and promoted angiogenesis in Src-dependent fashions^21^. These reports are consistent with our results (Figs 4 and 5) that collectively suggest that exosomes released from cancer cells can activate PI3K/Akt and MAP kinase signalling pathways in endothelial cells as well as promote angiogenesis *in vitro*. Moreover, Kawamoto *et al*. demonstrated uptake of tumour-derived microvesicles into the vascular endothelial cells via endocytosis with a dynasore reagent^13^. We demonstrated that dynamin-dependent endocytosis, such as clathrin-mediated endocytosis and/or caveolin-mediated endocytosis, was involved in the uptake of exosomes into endothelial cells (Figs 2, 4 and 5), which agrees with some previous studies^23^. These results suggested that endothelial cells actively take up exosomes in the extracellular spaces and use them as tools for cell-to-cell communications.

We indicated the predicted molecules of activated/inhibited signalling in HUVECs treated with exosomes released from PK-45H cells using the upstream analysis of the IPA system (Table 1). The functions of VEGF-A are to promote blood vessel dilation and permeability as well as to induce new blood vessel formation^24^. VEGF-A is overexpressed in most tumours, and the circulating levels of VEGF-A generally are elevated in cancer patients. VEGF receptor-2 (VEGFR-2) is expressed primarily in endothelial cells (Supplementary Fig. S3) consisting of seven extracellular immunoglobulin-like domains, a transmembrane region and an intracellular consensus tyrosine kinase domain. These domains activate the PI3K/Akt and MAP kinase signalling molecules, such as AKT, RAS and ERK1/2 and activators, such as PDGF BB^25^ and TRAF6^26^ (Table 1). U0126, which is predicted as ‘Inhibited’, is the MEK inhibitor of upstream molecules in ERK1/2^27^. As activated cancer-related or angiogenesis-related molecules, VEGF-A, HSP90^28^ and EDN1^29^ were predicted (Table 1). Sulforaphane, which is predicted as ‘Inhibited’, is known as an angiogenesis inhibitor^30^. Thus, we confirmed the activation of Akt and ERK1/2 as described above (Fig. 4). In the same manner, we believe that these signalling pathway predictions can be validated in HUVECs treated with exosomes released from PK-45H cells.

Exosomes are extracellular vesicles 40–200 nm in diameter, have small RNAs inside and are synthesised by an endosomal sorting complex required for transport (ESCRT)-dependent and/or ESCRT-independent pathway by lipid, such as ceramide and cholesterol^31^. Tetraspanins, such as CD63, CD9 and CD81, are known as surface markers of exosomes. Figure 1 shows that collected particles were on average 115 nm in diameter and had CD63 and small RNAs, suggesting the characteristics as exosomes. Therefore, exosomes released from PK-45H cells could be collected in this study.

Exosomes include several signal-inducer proteins and microRNAs inside and on the exosomal surface, and they induce functional changes in endothelial cells^17,19,32–34^. Nazarenko *et al*. indicated that exosomal tetraspanin Tspan8 derived from pancreatic adenocarcinoma cells contributed to activations of molecular pathways in endothelial cells and thereby enhanced proliferation, migration and tube formation^17^. Svensson *et al*. reported that a tissue factor into microvesicles released from hypoxia glioblastoma induced protease-activated receptor 2 activation in endothelial cells, increased phosphorylation of ERK1/2 and enhanced cell proliferation and migration^19^. On the other hand, Umezu *et al*. indicated that exosomal miR-135b derived from melanoma cell lines enhanced endothelial tube formation under hypoxia via the hypoxia-inducible factor-1α signalling pathway^34^. These reports suggest that specific exosomal components derived from cancer cells promote angiogenesis in endothelial cells. In addition, Zhou *et al*. and Taverna *et al*. observed that exosomal miR-105 and miR-126 released from cancer cells break down the tight junction between endothelial cells, respectively^32,33^. They suggested that exosomal microRNAs released from cancer cells may be directly involved in the metastasis of cancer cells and in engraftment of distant organs. The angiogenesis-inducer molecules in exosomes released from PK-45H cells remain unidentified. In the future, it is necessary to clarify inducer molecules of the PI3K/Akt and MAP kinase signalling pathways in HUVECs treated with exosomes released from PK-45H cells by metabolome and transcriptome analyses.

## Methods

### Cells and culture

The human pancreatic cancer cell line PK-45H (RCB1973) derived from liver metastasis was kindly provided by the Riken BRC as a part of the National Bio-Resource Project of the Ministry of Education, Culture, Sports, Science and Technology (MEXT), Japan. HUVECs were purchased from PromoCell GmbH (Heidelberg, Germany). PK-45H cells were cultured in RPMI-1640 medium (Wako, Tokyo, Japan) supplemented with 10% foetal bovine serum (Thermo Fisher Scientific), 100 U/mL penicillin and 100 µg/mL streptomycin (Wako). HUVECs were cultured in Endothelial Cell Media 2 (PromoCell GmbH) at 37 °C under a humidified atmosphere with 5% CO_2_.

### Purification and detection of exosomes

PK-45H cells were plated on 10 cm dishes at a density of 2 × 10^5^ cells/dish in the aforementioned culture media. After 4 days, the culture media were discarded, cells were washed thrice in serum-free culture medium and 10 mL serum-free culture medium was added to each dish. After 48 h, the cell culture media were collected and exosomes were isolated by a multistep centrifugation protocol. Exosomes from cell culture media were differentially centrifuged and purified using a procedure reported previously^35^. The collected exosomes were suspended in Dulbecco’s phosphate-buffered saline (D-PBS[−]). NanoSight LM10 (NanoSight) was used to detect the exosomes released from PK-45H cells.

### Western blotting

Protein extractions and Western blotting were performed using a procedure reported previously^35^. The primary antibodies used were as follows: Anti-CD63 (8A12) mouse monoclonal antibody (Cosmo Bio, Tokyo, Japan), anti-β actin rabbit polyclonal antibody (#ab8227; Abcam, Cambridge, UK), anti-GAPDH rabbit polyclonal antibody (#ab9485; Abcam), anti-RAB5 (C8B1) rabbit monoclonal antibody (#3547, Cell Signaling Technology [CST], Danvers, MA, USA], anti-phospho-Akt (Ser473; D9E) rabbit monoclonal antibody (#4060; CST), anti-Akt (C67E7) rabbit monoclonal antibody (#4691; CST), anti-phospho-ERK1/2 (Thr202/Tyr204) rabbit monoclonal antibody (#4370; CST) and anti-ERK1/2 (137F5) rabbit monoclonal antibody (#4695; CST). The secondary antibodies used were an anti-rabbit immunoglobulin G (IgG) horseradish peroxidase (HRP)-linked antibody (#7074; CST) and anti-mouse IgG HRP-linked antibody (#7076; CST). The bound antibodies were visualised with the ImmunoStar Zeta or LD Chemiluminescence System (Wako). To release antibodies binding on membranes, Stripping solution (Wako) was used according to the manufacturer’s instructions.

### RNA isolation and detection

Total RNAs were isolated using the Isogen II (NipponGene). The sizes of cellular and exosomal RNAs collected from the PK-45H cells were examined using the Agilent 2100 Bioanalyzer and Agilent RNA 6000 Pico Kits (Agilent Technologies)^36^. Total RNAs from HUVECs treated with exosomes were subjected to microarray analysis, as described below.

### Flow cytometry

HUVECs were seeded on 6–well plates at a density of 2 × 10^5^ cells/well in the Endothelial Cell Media 2. After 24 h, the culture medium was discarded and HUVECs were washed thrice in D-PBS(−). Then, the Endothelial Cell Media 2 was supplemented with dynasore (a dynamin inhibitor; Sigma-Aldrich, St. Louis, MO, USA) and added to each dish. Exosomes stained by the SYTO RNA Select (Thermo Fisher Scientific) were added to the culture medium of dynasore-treated or untreated HUVECs. After 3 h, the effects on the uptake of exosomes into HUVECs were examined using the BD FACS Aria.

### Visualisation of exosomes taken up into HUVECs

HUVECs were seeded in 8–well chamber slides at a density of 2 × 10^4^ cells/well. After 24 h, the slides were washed thrice in D-PBS(−), and the Endothelial Cell Media 2 containing exosomes stained by the SYTO RNA Select was added into each well, with or without dynasore. HUVECs were cultured for 3 h at 37 °C under a 5% CO_2_ humidified atmosphere. After 3 h, HUVECs were treated with 4% paraformaldehyde solution at room temperature for 20 min. After staining of the nuclei using the ProLong Gold Antifade Reagent with 4′,6–diamidino-2–phenylindole (DAPI; Thermo Fisher Scientific), the slides were covered with coverslips and visualised under a confocal laser scanning microscope (LSM710; Carl Zeiss, Oberkochen, Germany).

### Microarray analysis

The mRNAs in HUVECs treated with 10 ng/µL exosomes for 48 h and control HUVECs were labelled using the Low Input Quick-Amp Labeling Kit, One-colour (Agilent Technologies). The SurePrint G3 Human GE Microarray (8 × 60 K) slides (Agilent Technologies) were hybridised with cyanine-3 (Cy3)-labelled cRNA in a hybridisation solution prepared with the Gene Expression Hybridization Kit (Agilent Technologies) and incubated at 65 °C for 17 h using a hybridisation oven, as per the manufacturer’s instructions. The microarray slides were washed using the Gene Expression Wash Pack buffer (Agilent Technologies). Cy3 fluorescence signal images were obtained on the slides using the SureScan Microarray Scanner (Agilent Technologies) and processed using the Feature Extraction software (version 10.7), based on Agilent Technologies instructions. The expression data were processed by using GeneSpring GX14.5 software (Agilent Technologies). Increasing and decreasing probes of >1.5–fold at exosome treatment against no treatment were selected. IPA (Qiagen) was used to predict the change in function in HUVECs treated with exosomes released from PK-45H cells.

### Tube formation assay

Tube formation assay was performed using the Cultrex *In Vitro* Angiogenesis Assay Tube Formation Kit (Cosmo Bio). BME reagent (50 µL) was added to 96–well, flat-bottomed microplates and incubated at 37 °C for 60 min under a 5% CO_2_ humidified atmosphere. HUVECs then were seeded at a density of 1 × 10^4^ cells/well and treated with 10 ng/µL exosomes for 9 h in the presence or absence of 20 μM dynasore, 5 μM LY294002 or 0.5 μM U0126. Total branching length (mm) was monitored by light microscopy and measured using Image J (https://imagej.nih.gov/ij/) provided by the National Institutes of Health, Bethesda, MD, USA).

### Statistical analysis

Statistical analysis was performed using Excel 2010 (Microsoft, Redmond, WA, USA) with the add-in software Statcel 3 (OMS Publishing, Inc., Saitama, Japan) and the OriginPro 2018 software package (OriginLab, Northampton, MA, USA). Statistical significance (*P* ≤ 0.05) was examined by the Tukey–Kramer test for multiple comparisons.

## Electronic supplementary material

Supplementary information

Supplementary Table S1

Supplementary Table S2

Supplementary Table S3
