# Supplementary material for: Exosomes released from pancreatic cancer cells enhance angiogenic activities via dynamin-dependent endocytosis in endothelial cells in vitro
Source: Sci Rep. 2018 Aug 10;8:11972. doi: 10.1038/s41598-018-30446-1 (PMC6086824; doi:10.1038/s41598-018-30446-1)
Supplement: Supplementary file 1 — Supplementary information [file 41598_2018_30446_MOESM1_ESM.pdf]

Supplementary information

Exosomes released from pancreatic cancer cells enhance angiogenic activities via dynamin-dependent endocytosis in endothelial cells *in vitro*

Mitsuru Chiba[1,*], Shiori Kubota[2], Konomi Sato[2] and Satoru Monzen[3]

[1] Department of Bioscience and Laboratory Medicine, Graduate School of Health Sciences, Hirosaki University, 66–1 Hon-cho, Hirosaki, Aomori 036–8564, Japan

[2] Department of Medical Technology, School of Health Sciences, Hirosaki University, 66–1 Hon-cho, Hirosaki, Aomori 036–8564, Japan

[3] Department of Radiation Science, Graduate School of Health Sciences, Hirosaki University, 66–1 Hon-cho, Hirosaki, Aomori 036–8564, Japan

*Corresponding author: Mitsuru Chiba, Ph.D.
Department of Bioscience and Laboratory Medicine, Graduate School of Health Sciences, Hirosaki University, 66–1, Hon-cho, Hirosaki, Aomori 036–8564, Japan
E-mail: mchiba32@hirosaki-u.ac.jp

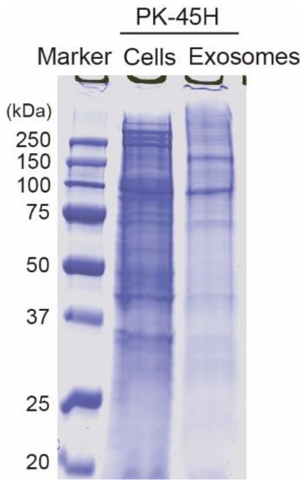

Supplementary Figure S1. Detection of cellular and exosomal proteins from PK-45H cells. Cellular and exosomal proteins (10 µg) from PK-45H cells in the loading gel were stained with Bio-Safe Coomassie brilliant blue (CBB) G250 Stain (Bio-Rad) according to the manufacturer's instructions. Stained gels were photographed using a GS-800 Calibrated Densitometer (Bio-Rad) and Quantity One software (Bio-Rad). Protein bands were detected in the exosomes.

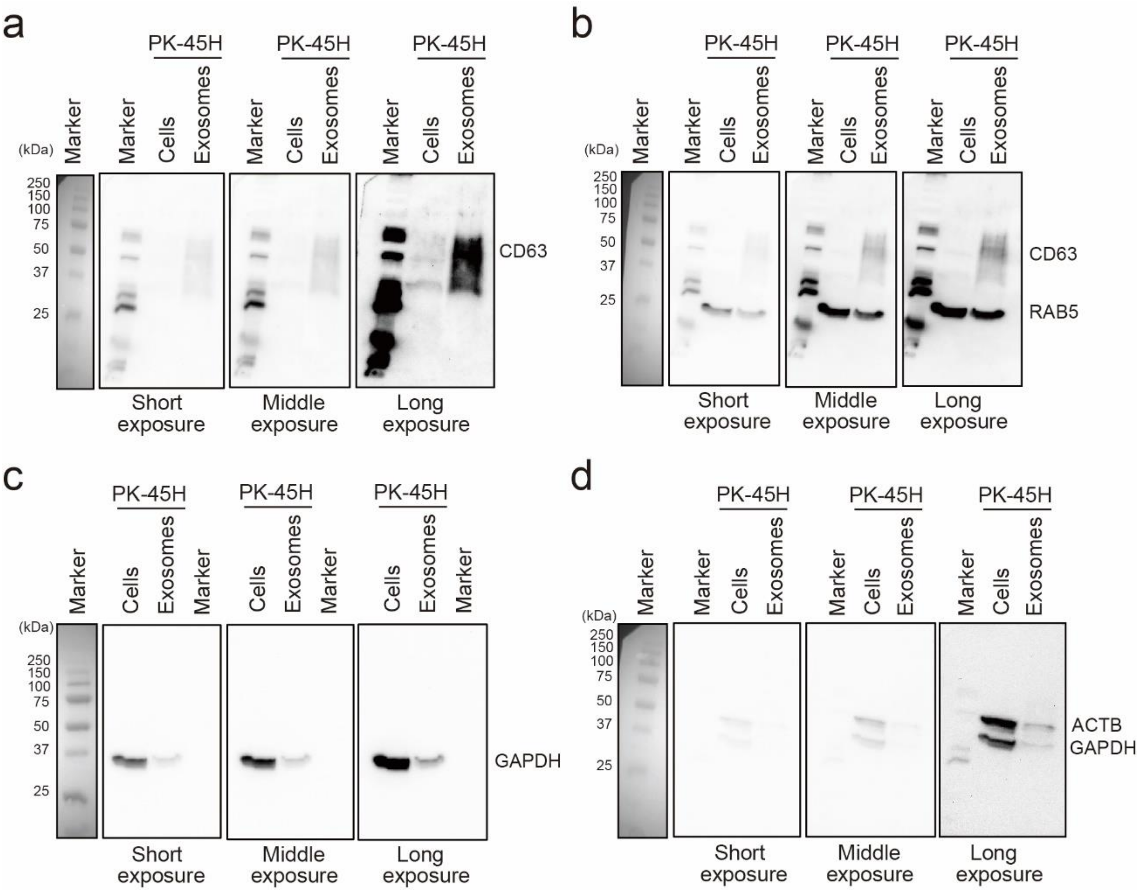

Supplementary Figure S2. The full-length blots of Fig. 1b. Detection of (**a**) CD 63, (**b**) RAB5, (**c**) GAPDH, (**d**) ACTB proteins in exosomes released from PK-45H cells and the cellular proteins by western blotting. To release antibodies binding on membranes, Stripping solution (Wako) was used according to the manufacturer's instructions. The membrane of (**a**) CD63 treated with Stripping solution was used to detect (**b**) RAB5. The membrane of (**a**) and (**b**) is the same. Likewise, the membrane of (**c**) GAPDH treated with Stripping solution was used to detect (**d**) ACTB. The membrane of (**c**) and (**d**) is the same. To optimally visualize all detected proteins, the exposure time was varied (short exposure, middle exposure and long exposure). Over-exposure of blots was not performed. The pictures near bands on membranes under long exposure were horizontally cut (Fig. 1b).

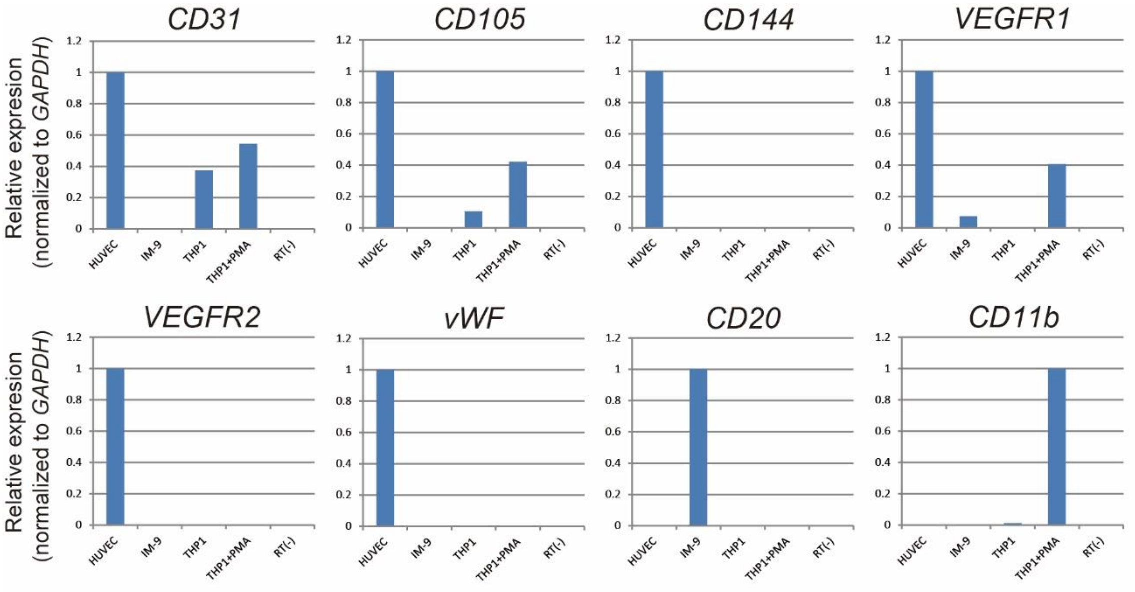

Supplementary Figure S3. Detection of endothelial markers in HUVECs. The detection of mRNAs in HUVECs, human B lymphoblast cells (IM-9) and human monocyte (THP-1) was performed by reverse-transcription quantitative polymerase chain reaction (qPCR). The cDNAs from total RNAs isolated from HUVECs were synthesised by using the High Capacity cDNA Reverse Transcriptase Kit (ThermoFisher Scientific), according to the manufacturer's instructions. The qPCR was performed using the Power SYBR Green (2×; ThermoFisher Scientific), 10.0 μM forward and reverse primers (Supplementary Table S4) and the StepOne Plus real-time PCR System (ThermoFisher Scientific), under the following conditions: 10 min at 95 °C, followed by 40 cycles at 95 °C for 15 s and 60 °C for 60 s. *GAPDH* was used as an internal control. The expression levels were determined by using the comparative Ct method. Here, mRNAs of endothelial markers (*CD31*, *CD105*, *CD144*, *VEGFR1*, *VEGFR2* and *vWF*), B cell marker (*CD20*), monocyte marker (*CD11b*) and *GAPDH* were examined. Primer sets used is given in Supplementary Table S4. HUVECs expressed the mRNAs of *CD31*, *CD105*, *CD144*, *VEGFR1*, *VEGFR2* and *vWF*. Phorbol 12-myristate 13-acetate (PMA) was used for induction of differentiation in THP-1.

Supplementary Table S4. Primer sequences for qPCR

Primer name	Sequences	Size (mer)	PCR products (bp)
CD31 forward primer	GCAACACAGTCCAGATAGTCGT	22	74
CD31 reverse primer	GACCTCAAACTGGGCATCAT	20	
CD144 forward primer	GCAGTCCAACGGAACAGAA	19	76
CD144 reverse primer	CATGAGCCTCTGCATCTTCC	20	
VEGFR1 forward primer	CCACCATCTGAACGTGGTTA	20	80
VEGFR1 reverse primer	TGCAGTATTCAACAATCACCATC	23	
VEGFR2 forward primer	GAACATTTGGGAAATCTCTTGC	22	66
VEGFR2 reverse primer	CGGAAGAACAATGTAGTCTTTGC	23	
CD105 forward primer	ACGCTCCCTCTGGCTGTT	18	88
CD105 reverse primer	GCTGAAGGTCACAATGGACTG	21	
vWF forward primer	GAAATGTGTCAGGAGCGATG	20	60
vWF reverse primer	ATCCAGGAGCTGTCCCTCA	19	
CD11b forward primer	GGCATCCGCAAAGTGGTA	18	70
CD11b reverse primer	GGATCTTAAAGGCATTCTTTCG	22	
CD20 forward primer	AGAACAAAATCTCTACTTTGATGGAAC	27	96
CD20 reverse primer	GGCAAGGCCTACTGCTGA	18	
GAPDH forward primer	AGCCACATCGCTCAGACAC	19	66
GAPDH reverse primer	GCCCAATACGACCAAATCC	19	

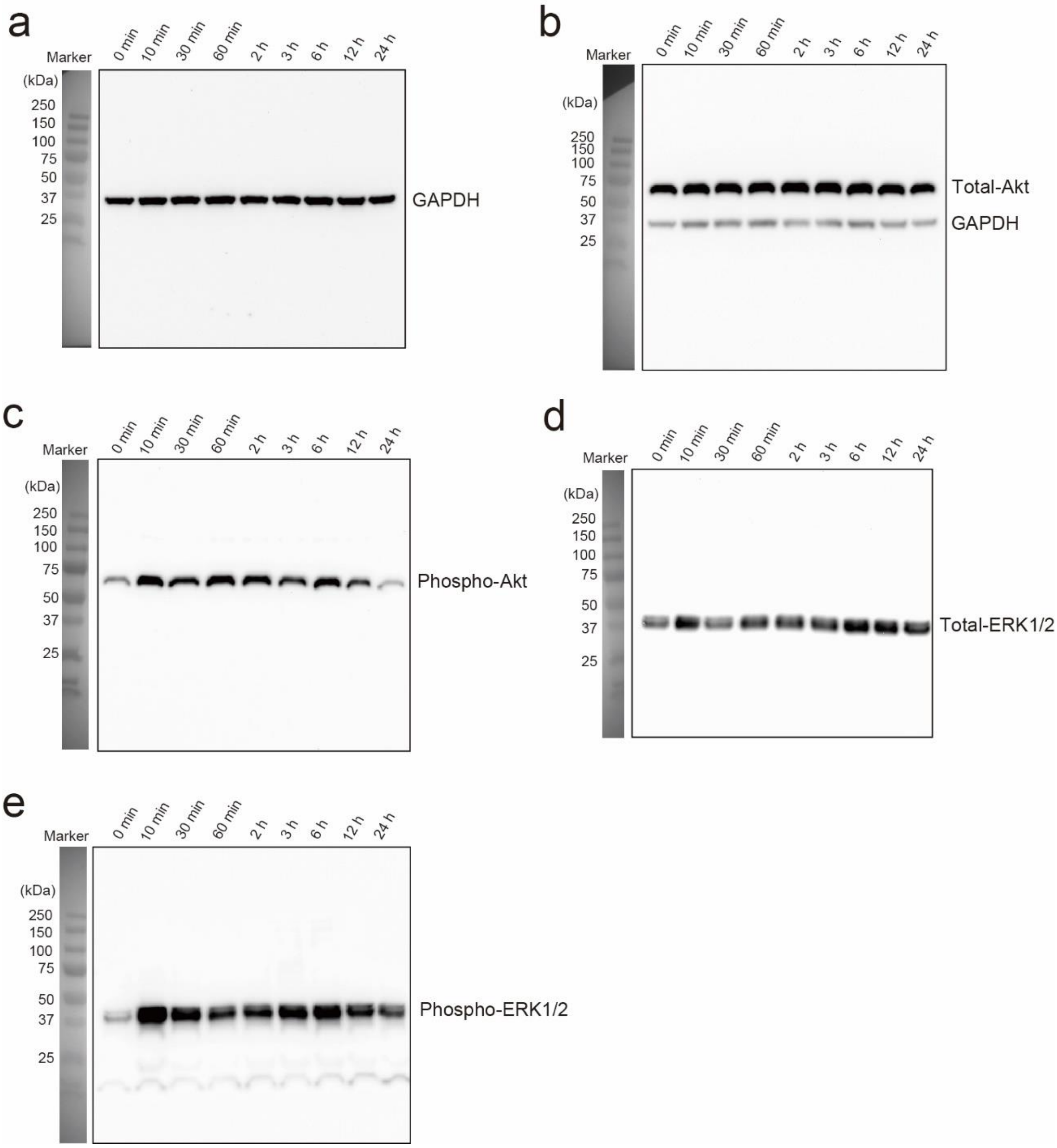

Supplementary Figure S4. The full-length blots of Fig. 4a. Detection of (**a**) GAPDH, (**b**) Total-Akt, (**c**) Phospho-Akt, (**d**) Total-ERK1/2, (**e**) Phospho-ERK1/2 proteins in HUVECs treated with exosomes released from PK-45H cells by western blotting. HUVECs were treated with exosomes for 0 min, 10 min, 30 min, 60 min, 2 h, 3 h, 6 h, 12 h and 24 h. Long exposure was performed as described in Supplementary Fig. S2. Over-exposure of blots was not performed. The membrane of (**a**) GAPDH treated with Stripping solution (Wako) was used to detect (**b**) Total-Akt. The membrane of (**a**) and (**b**) is the same, whereas of (**c**), (**d**) and (**e**) is different, respectively. The pictures near bands on membranes under long exposure were horizontally cut (Fig. 4a).

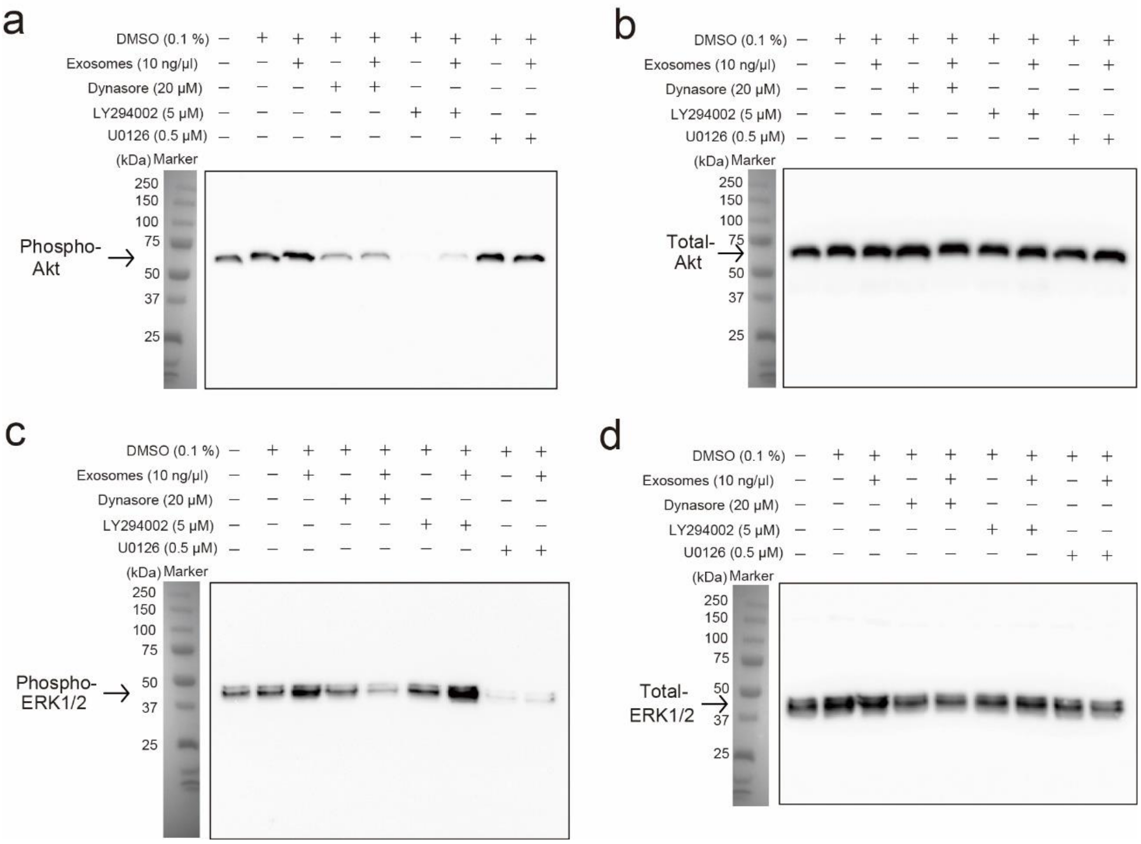

Supplementary Figure S5. The full-length blots of Fig. 4b. Detection of (**a**) Phospho-Akt, (**b**) Total-Akt, (**c**) Phospho-ERK1/2, (**d**) Total-ERK1/2 proteins in HUVECs treated with or without exosomes released from PK-45H cells in the presence or absence of dynasore, PI3 kinase inhibitor (LY294002) and MEK inhibitor (U0126) by western blotting. HUVECs were treated with exosomes for 10 min. Long exposure was performed as described in Supplementary Fig. S2. Over-exposure of blots was not performed. All membranes are different. The pictures near bands on membranes under long exposure were horizontally cut (Fig. 4b).

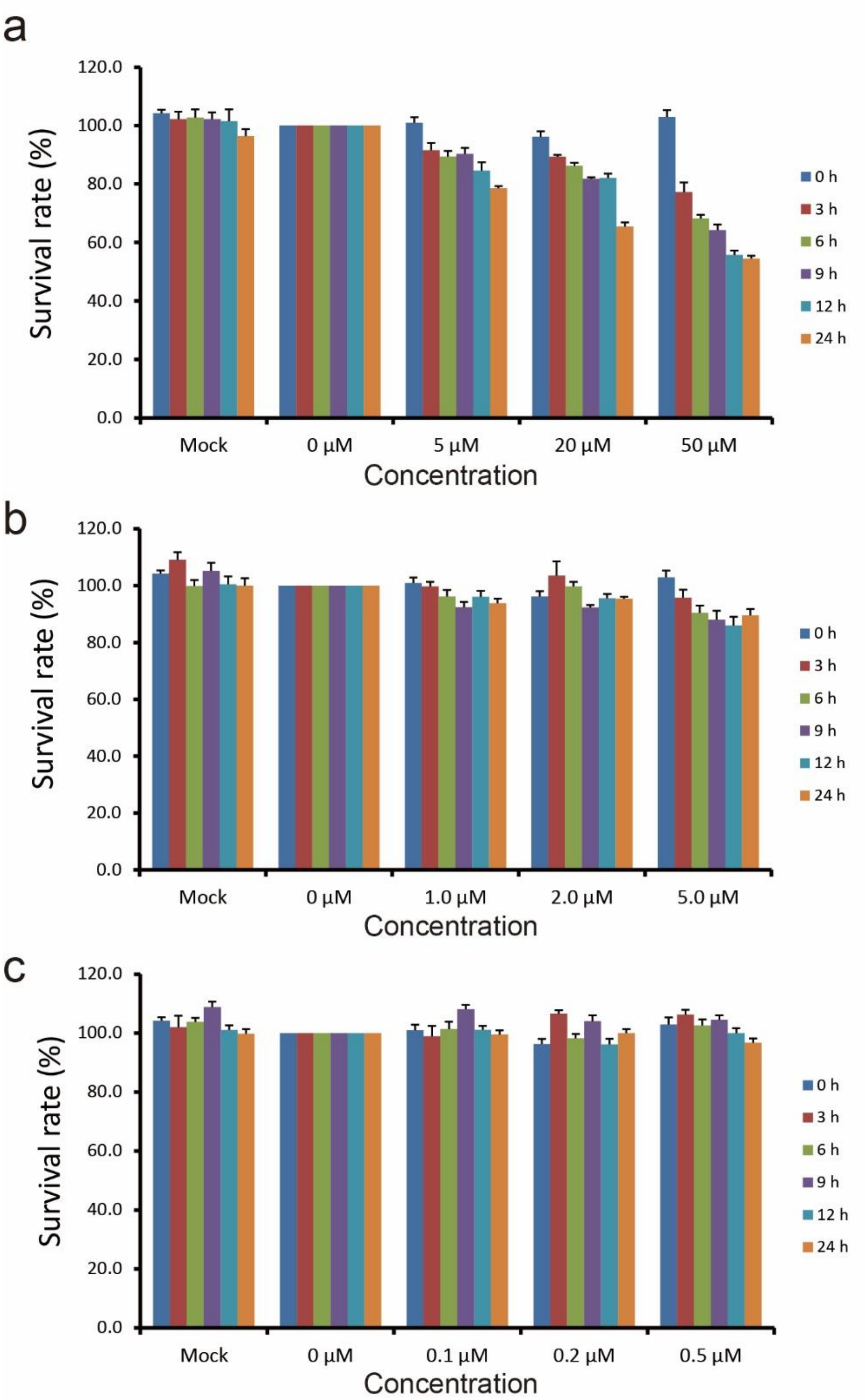

Supplementary Figure S6. Cell toxicity of HUVECs in the presence of dynasore, LY294002 and U0126. The cell proliferation assay in HUVECs was performed by using an alamar blue reagent (ThermoFisher Scientific) according to the manufacturer's instructions. The survival rate (%) of HUVECs treated with inhibitors, (**a**) Dynasore, (**b**) LY294002 and (**c**) U0126 was analysed. The data are expressed as means ± standard deviations (each $n = 8$).
